# Blind Depth-variant Deconvolution of 3D Data in Wide-field Fluorescence Microscopy

**DOI:** 10.1038/srep09894

**Published:** 2015-05-07

**Authors:** Boyoung Kim, Takeshi Naemura

**Affiliations:** 1 Graduate School of Information Science and Technology, The University of Tokyo, Tokyo, Japan

## Abstract

This paper proposes a new deconvolution method for 3D fluorescence wide-field microscopy. Most previous methods are insufficient in terms of restoring a 3D cell structure, since a point spread function (PSF) is simply assumed as depth-invariant, whereas a PSF of microscopy changes significantly along the optical axis. A few methods that consider a depth-variant PSF have been proposed; however, they are impractical, since they are non-blind approaches that use a known PSF in a pre-measuring condition, whereas an imaging condition of a target image is different from that of the pre-measuring. To solve these problems, this paper proposes a blind approach to estimate depth-variant specimen-dependent PSF and restore 3D cell structure. It is shown by experiments on that the proposed method outperforms the previous ones in terms of suppressing axial blur. The proposed method is composed of the following three steps: First, a non-parametric averaged PSF is estimated by the Richardson Lucy algorithm, whose initial parameter is given by the central depth prediction from intensity analysis. Second, the estimated PSF is fitted to Gibson's parametric PSF model via optimization, and depth-variant PSFs are generated. Third, a 3D cell structure is restored by using a depth-variant version of a generalized expectation-maximization.

3D wide-field fluorescence microscopy (WFM) is an essential tool in many disciplines, particularly biological and medical sciences. WFM provides molecular specificity by visualizing only biomolecules where fluorescence dyes can be selectively responded under a dark background. This property makes it possible to obtain micrographs with high contrast. Applying 3D WFM to observe 3D cellular structures refers to optical sectioning, that is, generating a series of discrete 2D image planes (*x*-*y* plane)^1^.

3D WFM, however, has several issues, such as out-of-focus blur obscuring the entire in-focus detail and thereby reducing the contrast of the in-focus object. Two major approaches to overcome these problems have been devised^1^. The first approach is to apply new microscopy optics. Confocal microscopy, the most widely used approach, suppresses out-of-focus blur by means of a pinhole. On the other hand, it causes limitations of slow image acquisition and photobleaching. The second approach is to apply image restoration by a deconvolution algorithm. It enhances the resolution and contrast of blurred WFM images that do not have any limitation mentioned above in the first approach. In this study, the second approach was focused on, and a method for deconvolution of 3D WFM images is proposed.

To implement the proposed image deconvolution algorithm, it is most important to obtain an accurate point spread function (PSF) of a 3D WFM imaging system. One of the main characteristics of a PSF of 3D WFM is depth variance along the optical axis (*z* axis), while general camera model ignores this variance^2^. This characteristic is because an aberration of WFM is caused by mismatch between the refractive indices of the immersion medium and the specimen. As the optical system focuses on a deeper specimen, the aberration increases. This aberration phenomenon is the mechanism of the depth-variant characteristics for 3D WFM.

Aiming to improve the resolution and contrast of 3D WFM through image deconvolution, numerous studies have been carried out^3^. Most of them have conducted depth-invariant image restoration owing to a simplicity of PSF modelling^4^,^5^,^6^,^7^. If the specimen is thin enough, the depth variance of PSF can be ignored, and their methods suppress the blur effectively, thereby increasing the resolution of 3D WFM up to that of confocal microscopy^8^. However, in case of an average size of common specimen (10–20*μm*), the axial blur along the *z* axis still remains^9^. For instance, the diameter of the blurred image of a 2500*nm* bead was measured as 4760*nm* (with axial blur) and 2867*nm* (with transverse blur), and after deconvolution of these values under the assumption that the specimen is thin enough, these results were respectively 4000*nm* and 2664*nm*^10^. These deconvoluted values indicate that the restored image is lengthened along the optical axis. This phenomenon, called elongation, occurs when the image is restored by using a depth-invariant PSF which is only suitable for a specific plane^2^. In consideration of the fact that the general size of an animal cell is 10–20*μm*, namely, much thicker than the 2500*nm* bead, the depth variance of PSF cannot be neglected. To handle the elongation problem, several researches have considered a depth-variant PSF. However, they tried experiments accompanied with pre-PSF measurement^2^,^11^ or only simulation^9^,^12^.

As for the pre-PSF measurement performed in previous studies, it was assumed that an actual PSF can be approximated by the captured 3D image of a point-like micro-bead. However, in the process of replacing a micro-bead with a cell specimen, the actual imaging condition including the optical path changes. This change makes the difference between the actual PSF and the result of pre-PSF measurement. In addition, a point-like micro-bead is merely a ‘point-like’ sphere and cannot be a perfect point source. For the above-mentioned reasons, the PSF should be estimated directly from captured images without any pre-measurement. This study focused not on simulation but on a more practical method that can be applied to a raw image and does not involve any pre-experiments for obtaining PSF information.

In order to evaluate and compare quantitative performances for actual WFM image, this paper uses the WFM observation data of precisely 2500*nm* diameter hollow sphere fluorescence micro-bead. Its diameter and shape becomes a ground truth. The diameter and the relative contrast between shell and sphere inside would be quantitative performance indicators^10^,^7^. Also, since the data is open to public, it enables comparing performances with the same data. Previous non-blind approaches evaluated their quantitative performances using simulation experiments^2^,^11^,^9^,^12^. Because they know the ground truth, they could use widely used performance indicators in general image restoration field such as mean square error, correlation coefficient values and so on. In some blind approaches, they evaluated their quantitative performances with simulation image that are generated by arbitrary PSF due to lack of ground truth image^13^,^14^. However, since the simulation image is generated under their assumption such as depth-invariant or symmetric PSF that are different from real one, they could get advantageous deconvolution result and it cannot be connected to the quantitative performance in actual image. Moreover, each study implements experiments using different images and initial PSFs, which makes difficulty to compare performances. Therefore, we used the open data of actual WFM image and could compare quantitative performances of our algorithm with existing software and algorithm.

Our algorithm estimates depth-variant specimen-dependent PSF under the specimen homogeneity (x-y invariant PSF). Our algorithm first roughly finds the PSF for centre of object by intensity analysis of the observed image. Then the PSF is modified through Richardson Lucy algorithm so as to maximize the conditional probability of the observed image given the PSF. To generate depth-variant PSFs, the modified PSF is parameterized using maximum likelihood function. Finally, depth-variant PSFs for every single depth in observed image are generated adjusting the depth parameter. Using generated depth-variant PSFs, the true object is estimated by penalized RL algorithm.

The major contributions of this study are as follows. First, a new practical WFM image deconvolution algorithm that reflects the depth variance of a PSF and actual imaging conditions is proposed. Second, it showed remarkable experimental results compared to the existing studies, and showed that our the proposed algorithm solved the problem of elongation and improved axial resolution. Third, our system is superior to other methods with regard to reducing the computational time.

## Results

Datasets of the C. Elegans embryo cell and fluorescent micro-bead images were used in two experiments. The first experiment on cells aimed to show the applicability and the qualitative performance of the proposed algorithm for biological images. The second experiment on beads, applied the proposed algorithm to the images of a fluorescent micro-bead whose size and shape were given. It was thus possible to evaluate the performance of the proposed algorithm quantitatively by comparing its quantitative performance to those of three different deconvolution software packages (Huygens Pro, AutoDeblur, Deconvolution Lab) as reported by Griffa^10^ and another depth-invariant method by Soulez^7^. The datasets can be downloaded from the website of the Biomedical Imaging Group in EPFL (http://bigwww.epfl.ch/deconvolution).

### C. Elegans embryo cell

The dataset is the observation image of a C. Elegans embryo cell with a × 100, 1.4*NA* oil UPlanSApo objective. Enough image stacks should be taken to allow overall shape of a specimen to be observed. Unfortunately, the dataset did not satisfy this condition and bring artefacts on boundaries of the restored image. To avoid the boundary effect, a dataset that is pre-processed by a minimum filter is used (see Method section). The data cube used was composed of 672 × 712 × 216 voxels of size 64.5*nm* × 64.5*nm* × 200*nm*. The PSF size (*x* × *y* × *z*) was set to 151 × 151 × 57 voxels of size 64.5*nm* × 64.5*nm* × 200*nm*. After deconvolution, the restored image was cropped to the original volume 672 × 712 × 104. The dataset was composed of three stacks of images corresponding to three wavelengths. CY3 (red 634*nm*), FITC (green 531*nm*) and DAPI (blue 447*nm*) staining represented the point-wise spots of protein, microtubule filaments and chromosomes in the nuclei, respectively. Each wavelength image was processed separately.

**Figure 1:**
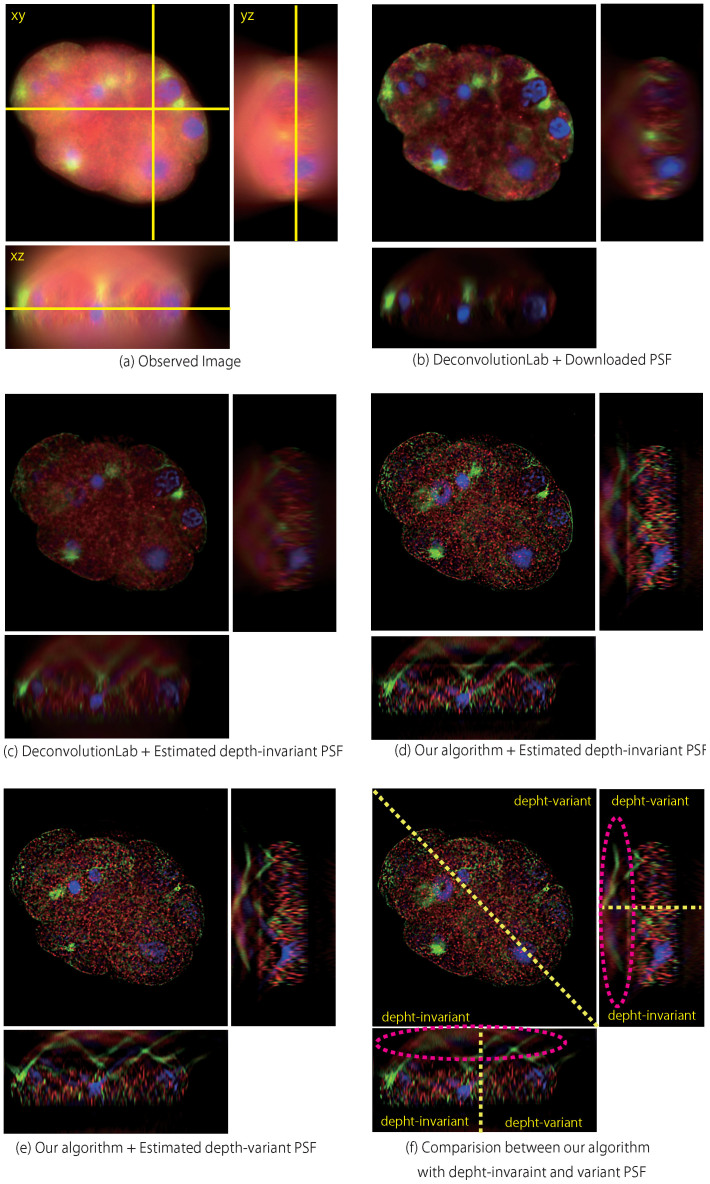
Result of image restorations by proposed algorithm and by DeconvolutionLab.

To compare the performance of the proposed algorithm with those of existing algorithms, the results of deconvolution by a commercial software package DeconvolutionLab as well as those obtained by the proposed deconvolution algorithm are depicted in Figure 1. All the experiments using ours and DeconvolutionLab were implemented with the same number of iterations 150. Table 1 summarizes the experimental conditions. The *x* – *y*, *y* – *z* and *x* – *z* profiles shown in Figure 1 are depicted when *z* = 63, *x* = 260 and *y* = 450 pixel, respectively. Performance of each algorithm was examined in terms of qualitative visibility and computational cost.

In raw data, the image detail is represented in a narrow intensity range. The acquired images corresponding to the CY3, FITC and DAPI channels have intensity ranges of (215–2842), (209–2929) and (206–2687), respectively. Each image was deconvoluted, the ranges were widened to (0–45898), (0–24773) and (0–16292), respectively.

An observed image of a C. Elegans embryo cell is shown in Figure 1(a). Since the image is blurry and unsharpened, it is difficult to identify its cellular components. A set of images restored by using DeconvolutionLab with a PSF downloaded from Biomedical Imaging Group in EPFL(http://bigwww.epfl.ch/deconvolution/?p=bio), which was generated without consideration of actual aberration, is shown in Figure 1(b). As shown in the figure, only components that had strong intensity remained, and even the remaining components are blurry. The result of image restoration using the depth-invariant PSF which was estimated in step 2 of the proposed algorithm (see Methods section) is shown in Figure 1(c). The result is still blurry, but it is improved from the viewpoint of observing specific components. It can be inferred from this result that the downloaded PSF did not reflect the actual imaging condition. The result obtained with the proposed accelerated generalized expectation-maximization (GEM) algorithm with the depth-invariant PSF is shown in Figure 1(d). The proposed algorithm had a clearer visibility than DeconvolutionLab after the same number of iterations since the image restoration was designed to guarantee the convergence and converge fast by means of vector extrapolation. The result of deconvolution by the proposed algorithm used depth-variant PSFs is depicted in Figure 1(e). While the restored image in Figure 1(d) is almost the same as that in Figure 1(e), the restored image in figure 1(f) shows that the elongation phenomenon was suppressed by our depth-variant GEM image algorithm. Moreover, it seems that the depth-variant GEM algorithm removed blur more effectively than the depth-invariant one, as represented in the pink elliptical area in Figure 1(f). When the observed C. Elegans embryo cell image in Figure 1(a) is compared with the restored image in Figure 1(e), it becomes clear that the proposed algorithm improves the visibility of the cellular structure. In addition, blue chromosomes, green filaments and red spots can be distinguished.

The processing time when DeconvolutionLab was used was five hours. The depth-invariant version of the proposed algorithm took only 113 minutes, which is obviously much faster than DeconvolutionLab. While the proposed depth-variant algorithm achieved better performance than that of the depth-invariant one in terms of qualitative visibility, it took more computational time (27.5 hours) than the depth-invariant version. In other words, a trade-off between performance and computational time exists.

### Fluorescent micro-bead

Observations of a InSpeck green fluorescent hollow bead with a diameter of 2500*nm* were used as a fluorescent micro-bead dataset. The observations were taken with an Olympus Cell R microscope with a × 63, 1.4 NA oil-immersion objective. The data cube was composed of 256 × 256 × 128 voxels with size 64.5*nm* × 64.5*nm* × 160*nm*. The PSF size (*x* × *y* × *z*) was set to 151 × 151 × 57 voxels of size 64.5*nm* × 64.5*nm* × 160*nm*.

The diameter of the restored image was measured in terms of the full width at half maximum (FWHM). As the FWHM value became closer to the real diameter (2500*nm*), the method was regarded as better one. The relative contrast between the border and the centre of the sphere used as a performance indicator because it was already known that the fluorescent bead was empty inside. As the relative contrast became higher, the boundary between the shell of the fluorescent bead and the hollow bead inside became more clearly distinguishable.

**Figure 2:**
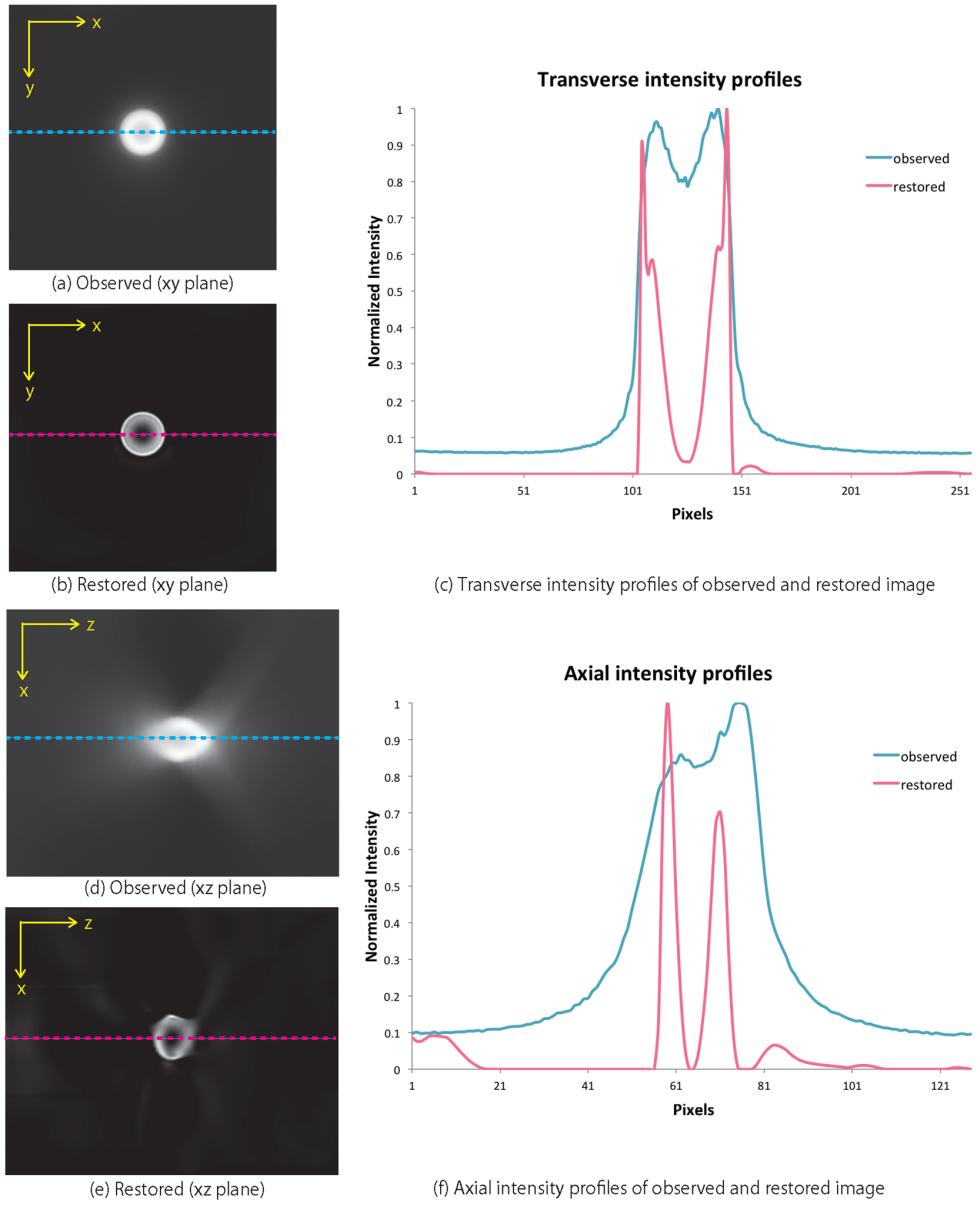
Blind depth-variant deconvolution results of 3D real fluorescence micro-bead images. Transverse intensity profiles in (c) are cuts along the blue dotted line in image (a) and the red dotted line in image (b). Axial intensity profiles in (f) are cuts along the blue dotted line in image (d) and the red dotted line in image (e).

Observed images and images restored by the proposed algorithm are shown in Figure 2. The images were normalized by dividing maximum intensity. Images observed along the transverse axis and the optical axis are shown in Figures 2(a) and (d), respectively. Images of a clear sphere shape restored from the ambiguous images in Figures 2(a) and (d) respectively are shown in Figures 2(b) and (e). Intensity profiles along the centre line (dotted line) in Figures 2(a) and (b) are plotted in Figure 2(c), in which the horizontal axis represents the position of the transverse axis. Blue and red lines depict the intensity of the observed and restored images, respectively. As can be seen from Figure 2(c), the border between the shell of the bead and the centre of the hollow sphere is definitely distinguishable. The relative contrast was calculated from the transverse intensity profiles in Figure 2(c). The axial intensity profiles shown in Figure 2(f) show the same tendency as those shown in Figure 2(c).

It is apparent from Figures 2(a) and (d) that the observed image is especially blurred along the optical axis in comparison to the transverse axis. As shown by the restored image and the intensity profile in Figures 2(e) and (f), respectively, the proposed algorithm clearly removed the blur along the optical axis. This result demonstrates that the elongation phenomenon was effectively suppressed. (The supplemental video shows the restoration process.).

To compare the quantitative evaluation, bead diameter error and relative contrast after previous deconvolution methods were applied to the images are listed in Table 2. As previously mentioned, the bead diameter was calculated as FWHM.

Parameter values of the observed image are presented in the ‘Raw data’ column. From the FWHM error values of raw data, it is clear that the blur was far severer along the optical than transverse axis. As the FWHM error of a deconvolution result gets closer to the zero, the deconvolution has better performance. As shown in Table 2, the axial FWHM error value given by the proposed algorithm was superior to those given by the other algorithms, which was closest to zero. This is because all of them except our algorithm assumed depth-invariant PSFs; thus, this result indicated the importance of applying depth-variant PSFs. The error in the axial FWHM value given by the proposed algorithm is 151*nm*, which is less than the voxel size along the optical axis (160*nm*). Although the error in the transverse FWHM value given by the proposed algorithm is 155*nm*, which is equivalent to 2.34 pixels on the transverse axis, the proposed algorithm gives the best transverse FWHM value. Besides, the relative contrast given by the proposed algorithm is also superior to those values given by the other algorithms. That is, the relative contrast given by the proposed method algorithm is 97%, and those values given by the other algorithms do not surpass 90%.

## Discussion

This study was undertaken to design a deconvolution algorithm for 3D WFM. Our proposed method removed axial blur effectively and solved the elongation problem via an accurate PSF estimation and a depth-variant image restoration. The proposed algorithm estimates a parameterized PSF reflecting actual imaging conditions from observed image, and it generates depth-variant PSFs controlling the depth parameter. A depth-variant image restoration algorithm, which is accelerated by vector extrapolation, was implemented. Results of the C. Elegans embryo cell and fluorescent bead experiment show that the proposed algorithm removes axial blur that could not be removed by algorithms developed in previous studies. Moreover, to compare quantitative performances of our algorithm with existing software and algorithm, we used the open dataset of 2500*nm* hollow sphere fluorescence bead. The quantitative performance values diameter error and relative contrast given by the proposed algorithm are superior to those given by a commercial software package used in this study. These findings suggest that 3D WFM images should be restored by a depth-variant deconvolution, and they imply that the PSF from an observation is more accurate than PSF measurement.

The bead used in this experiment is relatively thin and has about the size of bacteria. For very deep specimens, dataset generation of the object over 10 *um* would be worth for 3D deconvolution of WFM. Other possible directions for future work include fast algorithm for the depth-variant image restoration, *xyz* variant asymmetric PSF modelling and extending the proposed algorithm to other applications. The execution time for the proposed algorithm is discussed in the Results section, yet the algorithm does not operate in minutes. In our algorithm, simplex method for PSF parameter fitting and depth-variant convolution operator take most of processing time. Faster parameter fitting method and effective calculation such as distributed processing for depth-variant convolution operator would produce faster deconvolution method. According to the result of the experiment with fluorescent beads, the restored image has a shape of an asymmetric sphere. In this study, however, it was assumed that the PSF is *x*–*y* symmetric. The *xyz* asymmetric PSF would be a next task for the solution of the distorted result. Also, it is expected that not only *z* but also *xy* variant deconvolution would express inhomogeneity of specimen and improve accuracy of deconvolution result. Furthermore, the proposed algorithm can be also applicable to other image models that have a space-variant PSF such as astronomical image.

## Methods

The acquired 3D image, *g*, could be modelled by a 3D convolution between a 3D depth-variant PSF *h* and the true object *f* under noise model *n*:
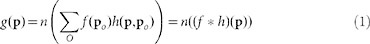


Where **p***_o_* = {*x_o_*,*y_o_*,*z_o_*} and **r***_o_* = {*x_o_*,*y_o_*} denote 3D and 2D object positions in object domain *O*, respectively. **p** = {*x*,*y*,*z*} and **r** = {*x*,*y*} are 3D and 2D image positions in image domain *I*, respectively. In this paper, the true object function illustrates the object in the air and does not depict image of the object in specimen layer. The PSF includes the object elongation effect due to refractive index mismatch. Since WFM images are taken in a dark room, the noise model of observed images follows a Poisson distribution^15^. As seen as eq (1), the PSF *h* varies according to positions of object **p***_o_* and image **p**. Since this paper ignores insignificant inhomogeneties in specimen layer^16^, the PSF variance along *x* and *y* axis (in one depth) is ignored. The aim of this study is to estimate the true object *f* from the acquired image *g*.

The proposed algorithm is composed of the following three steps: (1) estimation of a depth-invariant PSF, (2) generation of a depth-variant PSF and (3) restoration of a depth-variant image.

In step 1, a single non-parameterized PSF *h_step_*_1_(**p**) for an overall region is estimated. For constructing depth-variant PSFs, nonparametric *h_step_*_1_(**p**) is converted to parametric PSF model *h_step_*_2_(**r**−**r***_o_*,*z*;*z_o_*). Controlling the depth parameter *z_o_* makes it possible to obtain depth-variant PSFs. Depth-variant image restoration, which is accelerated by vector extrapolation, is then implemented^18^.

### Step 1. Estimation of depth-invariant PSF

In step 1, an initial PSF is estimated first. Before the procedure for estimating PSF is explained, the method for generating the initial PSF and its specific setting are explained. The accuracy of the estimated PSF depends on the initial PSF. To generate the initial PSF, the Gibson and Lanni PSF model, which is based on Kirchhoffs integral formula (one of the most widely used PSF models for WFM), was applied^16^. This model generates a 3D WFM PSF by substituting optical parameters. These parameters are refractive indices and optical distances, which are determined by analyzing the intensity profile and objective lens information. The Gibson and Lanni PSF model is given as
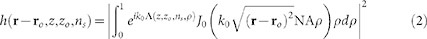
where *k*_0_ denotes the vacuum wave number, NA is the numerical aperture, and Λ(*z*,*z_o_*,*n_s_*,*ρ*) represents the optical path difference (OPD) between the design and actual conditions. *J*_0_ denotes the Bessel function of the first kind of the zero.

**Figure 3:**
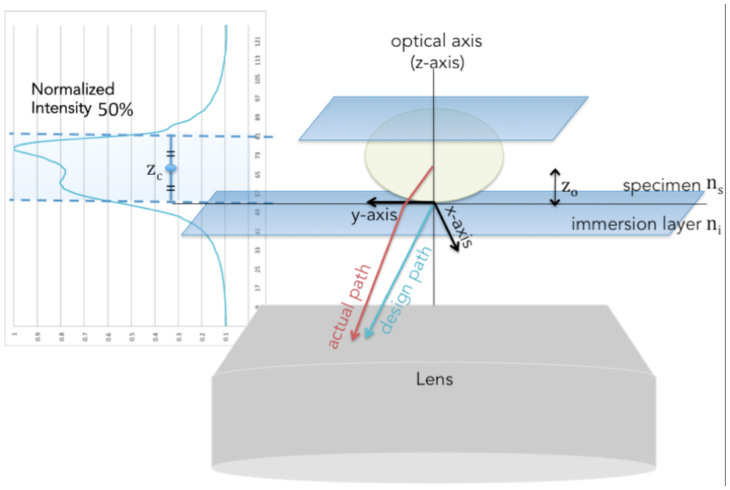
A schematic of optical path in WFM; the left graph illustrates the normalized intensity along the *z* axis to help understanding of initial parameter *z_o_*. When the initial parameter *z_o_* is set, the normalized intensity that is bigger than [(*min*(*g*(*z*))+*max*(*z*)))/2] in the centre of the *x* and *y* axes is supposed to be the object.

A schematic of the optical path in a WFM is shown in Figure 3. The OPD, Λ(*z*,*z_o_*,*ρ*), causes spherical aberration, which is modelled as^19^
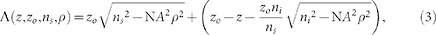
where *n_s_* and *n_i_* represent the refractive indices of the specimen and the immersion layer. Since the refractive index of internal cellular components (1.33–1.37) are usually similar to those of water, initial *n_s_* is set as the refractive index of water^17^. If user uses other types of samples such as glycerol solution, the refractive index value for initial PSF would be changed. Meanwhile *n_i_* depends on the composition of immersion layer. In the case an oil-immersion objective is used, *n_i_* is taken as the refractive index of oil.

Unknown parameter *z_o_* denotes the position of the object on the *z* axis. The initial *z_o_* setting is calculated from the intensity profile of the captured image. To make it easier to understand, setting of parameter *z_o_* is depicted in Figure 3. The object part is set as normalized intensities greater than [(*min*(*g*(*z*))+*max*(*z*)))/2] at the origin of the *x* and *y* axes. *Z_o_* is then set as the central position *z_c_* of the object part, under the assumption that the lowest position of the object part as *z_o_* = 0. Then the initial PSF, *h*(**r**−**r***_o_*,*z*;*z_o_* = *z_c_*,*n_s_*), is generated by using Equation (2)(3).

After the initial PSF is generated, a single PSF for the overall region is estimated. In this step, a non-parameterized and image-based PSF model is used, while the initial PSF is is derived from the parameterized equation. This is because the non-parameterized PSF estimation is quicker than parameterized PSF estimation.

The equation for finding the true object and the PSF that maximize the conditional probability of the observed image is given as
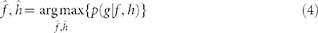


Since a WFM image follows a Poisson distribution, an objective function can be expressed as
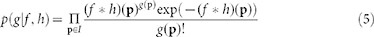
Due to difficulty in differentiating eq (5), the problem from maximizing eq (5) is changed to minimizing the following negative log-likelihood function:^20^
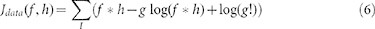
After eq (6) is differentiated with respect to *f* and *h*, the derivative is equated with zero to yield the following equation^21^:
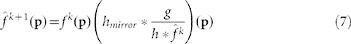
where *k* indicates the iteration number. *h_mirror_*(**p**) = *h*(−**p**) and *f_mirror_*(**p**) = *f*(−**p**) represent the mirrored PSF and true object, respectively. This equation is called a blind Richardson-Lucy (RL) algorithm, which is often used for deconvolution of the data in a Poisson distribution. The blind RL algorithm iteratively estimates the true object *f* and the non-parameterized PSF *h* simultaneously from the acquired image *g* and the initial PSF, *h*(**r**−**r***_o_*,*z*;*z_o_* = *z_c_*,*n_s_*). The initial *f* is the acquired image, *g*. In this step, however, the blind algorithm is utilized only for estimating the PSF. The estimated PSF, *ĥ*=*h*_*step*1_, is considered as the actual PSF corresponding to the centre of the object.

### Step 2. Generation of depth-variant PSF

To construct depth-variant PSFs from a non-parameterized model, it is required to estimate PSFs for each depth. That estimation, however, is difficult and takes a lot of computational time. If the PSF is converted to a parameterized model, depth-variant PSFs could be effectively generated by controlling parameter *z_o_*.

To do so, it is necessary to estimate *z_o_* and *n_s_* of Equation (2) that minimizes a negative log-likelihood of a given *h_step_*_1_(**p***_i_*) Poisson distribution.
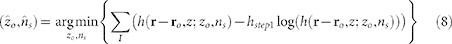
Equation (8) is minimized by a simplex method, which is a simple and fast mathematical optimization^22^. Iteratively, Equation (8) is implemented until convergence. A parameterized PSF, *h*_*step*2_(**r**−**r**_*o*_,*z*;*z_o_*,*n_s_*)=*h*(**r**−**r**_*o*_,*z*;*ẑ_o_*,*n̂_s_*), that reflects the position and the refractive index of the specimen can then be obtained.

**Figure 4:**
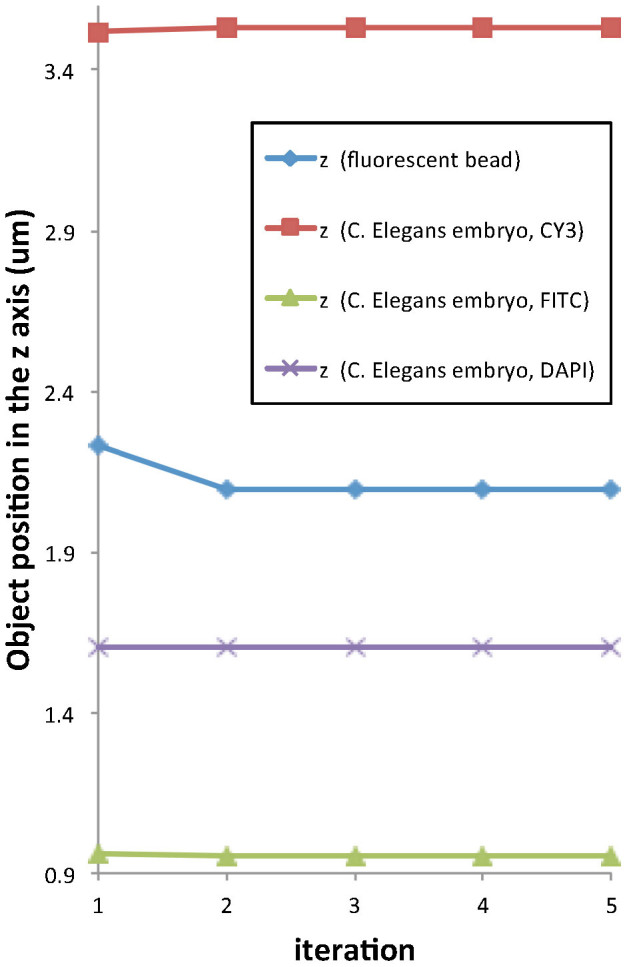
Stability of parameter *z_o_* optimization.

Specific parameter settings and parameter curves in the case of the above-described experiments with a fluorescent bead and C. Elegans embryo cell are described in the following. Since the datasets were taken by an oil-immersion lens, the refractive index of the immersion layer is set as *n_i_* = 1.518. Curves of parameter *z_o_* for PSF fitting are shown in Figure 4. In our bead and C.Elegans embryo cell experiments, the refractive index parameter *n_s_* showed no variation. It can be seen from the figure that the parameter-fitting procedure needs only few iterations and that the parameter curves all converge. In our experiments, the iteration was stopped if the parameter did not change three times in a row.

The PSF equation, namely, (2)(3), into which *z_o_*=*ẑ_o_* and *n_s_*=*n̂_s_* is substituted becomes the actual parameterized PSF for the central depth of the object. And then, depth-variant PSFs are generated by shifting parameter *z_o_* in accordance with the axial resolution of the acquired image.

### Step 3. Restoration of depth-variant image

In this step, a penalized depth-variant RL algorithm is used for restoring the depth-variant image. An image following a Poisson distribution is relatively weaker in respect to noise than a Gaussian distribution; thus, the penalized version of the RL algorithm^23^ was chosen. The penalized RL algorithm restores the image by maximizing the penalized likelihood function, defined as follows:
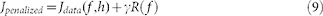
where *γ* is the regularization parameter. The total variation regularization constraint, which preserves edges due to its linear penalty on difference between adjacent pixels, was set as follows:
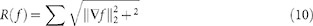
The regularization parameters were set as *γ*=0.5×10^−5^ and 0.1 × 10^−3^ for the cell and bead experiments, respectively.

**Figure 5:**
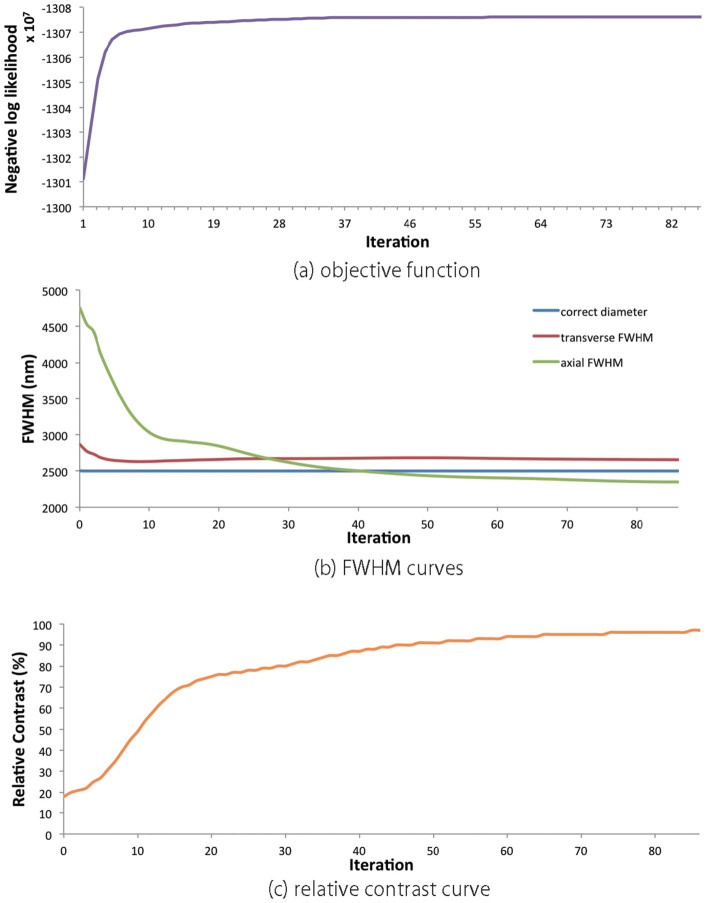
**(a) Objective function curve;** (b) transverse and axial FWHMs computed at each iteration; (c) relative contrast curve.

The total variation penalty couples each pixel in the restoration with its adjacent neighbours in such a way that a direct derivative for maximizing the penalized likelihood function is not possible^24^. As the means of solving this problem, most previous studies approximated the difference between adjacent pixels as the difference between the value of a current pixel and the values of the neighbouring pixels from the previous iteration^23^,^7^. However, a restored image using the approximation is not accurate since this method does not converge to the solution monotonically. The generalized expectation-maximization (GEM) algorithm was thus chosen to evaluate the derivatives indirectly by using the quadratic surrogates of the regularization term^25^. The final form of the depth-variant GEM algorithm is given as follows^11^:
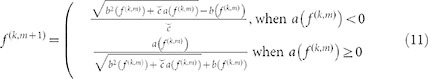
where *c̆* and *m* represent curvature and sub-iteration, respectively. *a*( *f* ^(*k,m*)^) and *b*( *f* ^(*k,m*)^) are defined as
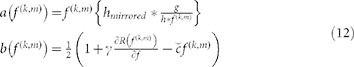
This iterative technique, however, is slow to converge toward the final result. To increase the speed of convergence, vector extrapolation is applied^18^. The acceleration method predicts where each pixel in the image is going from the correction obtained by each iteration. A new point *c^k^* is predicted, and the GEM algorithm is applied to generate the next estimate *f ^k^*^+1^ and gradient *d^k^* as follows:
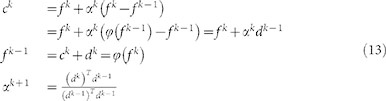
Changes in the objective function, FWHM and relative contrast value during iteration of image restoration of the fluorescent bead image are shown in Figures 5(a), (b) and (c), respectively. The objective function represents a negative log-likelihood function, which is calculated from Equation (9). The smaller the negative log-likelihood function, the more accurately the true object is estimated. In Figure 5(a), it is clear that the objective function converges enough. The iteration is stopped when the relative contrast and FWHM values does not change; accordingly, the results were obtained after 87 iterations. In Figure 5(b), the axial FWHM value changes rapidly for ten iterations, whereas the transverse FWHM curve changes smoothly. In Figure 5(c), the relative contrast rapidly increases in the early stage, namely, a similar tendency with the axial FWHM curve.

### Preparation of data set

To compare the quantitative performance of the proposed algorithm with that of other algorithms, it was tested by using the same data sets as those used in previous studies. The data sets are composed of stacks of images of a 2.5*μm* diameter fluorescent microbead and a C. Elegans embryo. In the case of the data sets for the C. Elegans embryo cell, not enough *z* stacks were taken to visualize whole shape of the object. To prevent the artefact occurring at the boundary, the data size was extended, and a minimum filter was applied to the extended area as follows.

First, after a 672 × 712 × 216 matrix was generated, the raw data of a C. Elegans embryo image (672 × 712 × 104) was put in the centre of the generated matrix (57–160 plane along *z* axis). Then, intensity values for the unfilled areas were determined by the minimum filter. Undetermined values bordering determined values were calculated as follows. The minimum values obtained by the minimum filter were found in the 3 × 3 matrix of neighbouring determined pixels, and the unfilled pixels right above or below the neighbouring determined pixels were filled in. In this way, the whole matrix was fully filled and could be used for the experiments. Through this procedure, enough *z* stacks could be obtained until most of the intensities along the *z* axis disappeared, thereby reducing artefacts at the boundary. After image restoration, the restored image was then cropped back to the same size as the raw image.

### Computational features

All procedures were carried out in MATLAB 2014a on parallel Intel Xeon E5-2680 processors (2.8 GHz) 448GB RAM, running Windows. Total computational time for the fluorescent bead experiment was about 265 minutes. step 1 took 5 minutes. The parameter estimation for PSF fitting and the depth-variant PSF generation in step 2 took 70 minutes and 120 minutes, respectively. Step 3 for image restoration took 70 minutes.

## Supplementary Material

Supplementary Information Supplemental Video S1

Supplementary Information Supplemental Video S2

Supplementary Information Supplemental Video S3

Supplementary Information Supplemental Video S4

Supplementary Information Supplementary Information

## Figures and Tables

**Table 1:** Experimental conditions for comparison

	Algorithm	PSF
(b)	RL (DeconvolutionLab)	PSF data from BIG
(c)		Depth-invariant PSF in step 2
(d)	GEM (Proposed algorithm)	Depth-invariant PSF in step 2
(e)		Proposed depth-variant PSF

**Table 2:** Performance comparison of previous^7^ and proposed methods

	Raw data	Huygens	AutoDeblur	DeconvLab	ISBI 2012	Proposed
Transverse						
FWHM	367	209	209	164	236	155
error (nm)						
Axial						
FWHM	2260	1500	2140	1660	477	151
error (nm)						
Relative						
	18	53	78	68	88	97
Contrast (%)						
