# Supplementary material for: Blind Depth-variant Deconvolution of 3D Data in Wide-field Fluorescence Microscopy
Source: Sci Rep. 2015 May 7;5:9894. doi: 10.1038/srep09894 (PMC5155489; doi:10.1038/srep09894)
Supplement: Supplementary Information [file srep09894-s5.pdf]

Supplementary Information

Blind Depth-variant Deconvolution of 3D Data in Wide-field Fluorescence Microscopy

Boyoung Kim[1,*] and Takeshi Naemura[1]

[1]*Graduate School of Information Science and Technology, The University of Tokyo, Tokyo, Japan*

[*] Correspondence should be addressed to B.K. (bykim@nae-lab.org).

Supplementary Videos

Supplementary Video S1: Blind depth-variant deconvolution result of 3D real fluorescence micro-bead after each iteration (transverse cut)

Supplementary Video S2: Blind depth-variant deconvolution result of 3D real fluorescence micro-bead after each iteration (transverse intensity profile)

Supplementary Video S3: Blind depth-variant deconvolution result of 3D real fluorescence micro-bead after each iteration (axial cut)

Supplementary Video S4: Blind depth-variant deconvolution result of 3D real fluorescence micro-bead after each iteration (axial intensity profile)
